# Telehealth Approaches for Pediatric Otitis Media and Clinical Outcomes: Scoping Review

**DOI:** 10.2196/85416

**Published:** 2026-03-18

**Authors:** Masao Noda, Ryohei Akiyoshi, Makoto Hosoya, Chikako Shinkawa, Ryota Koshu, Hidekane Yoshimura, Yukifumi Monden, Hiroaki Fushiki, Yasuhiro Tanaka, Makoto Ito

**Affiliations:** 1 Department of Otolaryngology and Head and Neck Surgery, Jichi Medical University, 3311-1, Yakushiji, Shimotsuke, 3290498, Japan, 81 0285442111; 2 Department of Otolaryngology and Head and Neck Surgery, Dokkyo Medical University Saitama Medical Center, Saitama, Japan; 3 Department of Otolaryngology and Head and Neck Surgery, Keio University, Tokyo, Japan; 4 Department of Otolaryngology and Head and Neck Surgery, Yamagata University, Yamagata, Japan; 5 Department of Otolaryngology – Head and Neck Surgery, Shinshu University School of Medicine, Matsumoto, Japan; 6 Department of Pediatrics, Jichi Medical University Hospital, Shimotsuke, Japan; 7 Department of Otolaryngology, Mejiro University Ear Institute Clinic, Saitama, Japan

**Keywords:** pediatrics, acute otitis media, otitis media with effusion, telemedicine, telehealth, smartphone otoscopy, digital otoscopy, artificial intelligence, tympanostomy

## Abstract

**Background:**

Otitis media (OM) is a common pediatric infection worldwide. Conventionally, accurate diagnosis depends on in-person pneumatic otoscopy, which is not always accessible, contributing to delayed care and inappropriate prescribing, especially in underserved settings. Rapid advances in telemedicine and digital tools have accelerated the development of remote approaches for assessing pediatric ear diseases, while diagnostic quality maintenance, care models, and real-world outcomes have not been comprehensively mapped in children.

**Objective:**

This study aimed to map existing telehealth technologies and operational models used for pediatric OM and report their diagnostic and implementation outcomes to guide practice and further research.

**Methods:**

PRISMA-ScR (Preferred Reporting Items for Systematic Reviews and Meta-Analyses extension for Scoping Reviews) guided this scoping review (protocol not registered). MEDLINE (via PubMed), Scopus, Web of Science, and the Cochrane Central Register of Controlled Trials were searched for original English-language studies (published between January 1, 2010, and February 9, 2026) reporting the clinical implementation of telemedicine or digital health in children (<18 years) with suspected or confirmed OM. We excluded reviews, editorials, and protocols; conference abstracts; adult-only studies; and purely technical evaluations. Two reviewers independently extracted and charted the study characteristics: telemedicine model, technology, users, comparators, outcomes, and limitations. Findings were synthesized using practice-oriented mapping that aligned technologies with care models and implementation conditions.

**Results:**

Fifty-two studies across 18 countries and various settings met the inclusion criteria. Sample sizes ranged from 6 to 3950, with heterogeneous reporting units (children, ears, episodes, or screening assessments). Asynchronous store-and-forward tele-otoscopy was the most common approach. The rest used synchronous or hybrid models. Image capture by trained personnel and review by experienced clinicians yielded substantial diagnostic consistency with in-person microscopy (κ 0.68‐0.89, sensitivity 72%‐94%, specificity 93%‐98%, where available). However, the diagnostic yield was highly dependent on the training level: structured instruction improved video capture by parents and nonspecialists, whereas brief or written-only guidance resulted in low rates of diagnostically useful videos. Telemedicine approaches improved access, supported perioperative follow-up, and, in some contexts, reduced reexaminations and promoted more judicious antibiotic use. Televisits without otoscopy were associated with lower confirmation rates of middle ear effusion during tympanostomy tube placement. Evidence was heterogeneous, with predominantly small single-site studies, variable reference standards and operator training, and rapidly evolving device ecosystems.

**Conclusions:**

This review provides a practice-oriented map of telehealth approaches for pediatric OM. Tele-otoscopy and adjunct digital tools are feasible, achieving diagnostic accuracy comparable to in-person assessments while enhancing access and service efficiency. However, important evidence gaps remain, including the need for large multisite trials, evaluation of long-term child outcomes, economic evaluations, and robust external validation of artificial intelligence–based diagnostic tools. Standardization of image capture protocols and integration into hybrid care models should be prioritized for scaling up.

## Introduction

Telemedicine has expanded rapidly across pediatrics over the past decade, further accelerated by the COVID-19 pandemic. Telehealth performance is disease specific, determined by whether robust remote proxies for the examination exist and are embedded in workflows [1,2]. In practice, the feasibility and safety of remote pediatric care depend on whether key physical findings can be reliably captured and shared. Equity concerns related to devices, connectivity, and digital literacy also underscore the need to track and address the digital divide [3]. For ear disease, this principally entails dependable tympanic membrane visualization and measures of middle ear status [4,5].

Within this context, otitis media (OM) is among the most common pediatric infections worldwide and a leading cause of health care visits, antibiotic prescriptions, and tympanostomy tube placement [6,7]. In 2021, the estimated global incident cases of OM in children were 297 million, highlighting the substantial population burden [6]. OM also contributes substantially to pediatric antibiotic exposure; a previous study reports that in a large outpatient cohort, antibiotics were prescribed in 44.8% of OM encounters [8]. Recent evidence from large outpatient datasets suggests that antibiotic prescribing patterns can differ between telemedicine and in-person visits for acute respiratory infections, raising concerns that diagnostic uncertainty—particularly when objective otoscopic findings are unavailable—may influence management [9]. Acute OM (AOM) and OM with effusion (OME) impose substantial morbidity, reduced quality of life, and increased health care costs across diverse settings [7,10], with a global review reporting annual costs as high as US $5 billion in the United States [10]. Accurate diagnosis hinges on direct visualization of the tympanic membrane and assessment of middle ear status—ideally by pneumatic otoscopy—which requires specific training and equipment [7,11]. Limited access to clinicians trained in pediatric otoscopy, particularly in rural and underserved regions, can contribute to delayed assessment and potentially inappropriate treatment [7].

Concurrently, digital health technologies relevant to ear disease have proliferated [12-14], including smartphone-connected and USB video-otoscopes, non-smartphone digital otoscopes, remote monitoring platforms, and early artificial intelligence–assisted decision support [15,16]. Beyond clinician-facilitated workflows, newer models increasingly rely on caregiver- or patient-captured otoscopic media for asynchronous review; however, interpretability can vary markedly depending on who captures the recordings, indicating that real-world deployment requires systems that standardize capture quality through clear thresholds, training, and feedback loops [17]. When paired with appropriate workflows, these tools enable asynchronous review by experts or real-time supervision and can be complemented by adjunct tests such as tympanometry or acoustic methods [18-23]. Emerging studies suggest that telemedicine can approximate in-person diagnostic performance for pediatric OM when image capture is standardized and operators are trained while also reducing barriers to care and supporting antibiotic stewardship [20,21,24]. However, important uncertainties remain regarding feasibility, usability, and sustainable integration into clinical practice.

Given the rapidly expanding yet fragmented evidence base, we conducted a scoping review to (1) map the technologies and care models used in telehealth for pediatric OM, (2) summarize diagnostic and implementation outcomes, and (3) identify evidence gaps to guide future research and clinical implementation. This review further aims to identify the key barriers and enablers of feasibility and to provide actionable guidance for implementing pediatric OM telehealth—across technologies, care models, and settings.

## Methods

### Overall Study Design

A scoping review was performed according to the PRISMA-ScR (Preferred Reporting Items for Systematic Reviews and Meta-Analyses extension for Scoping Reviews) checklist [25]. The review process progressed through the stages outlined in the framework of Arksey and O’Malley [26], which included formulating the research question; identifying relevant studies; selecting studies; extracting and organizing the data; and synthesizing, summarizing, and presenting the findings. The protocol for this scoping review was neither registered nor published. Core decisions were defined a priori (research questions, eligibility criteria, databases, and data items). Minor refinements to the screening guidance, including operational definitions of clinical implementation and tele-otoscopy, were made iteratively during pilot screening to improve consistency and were applied uniformly thereafter. In addition, reporting of the literature search was guided by the PRISMA-S statement (an extension to the PRISMA Statement for Reporting Literature Searches in Systematic Reviews) to enhance transparency and reproducibility of the search methods [27].

### Information Sources and Search Strategy

MEDLINE (via PubMed), Scopus, Web of Science Core Collection, and the Cochrane Central Register of Controlled Trials (CENTRAL) were searched for studies published between January 1, 2010, to February 9, 2026 (publication cutoff date), using a predefined strategy developed by 3 authors (MN, RA, and MH). Searches were last run on February 9, 2026, as the final rerun during the revision process; no publication date restrictions were applied beyond the search date, and no automated alerts were used. Each database was searched separately on its native platform rather than via a single multidatabase interface.

The strategy combined controlled vocabulary and free-text terms for three concepts: (1) otitis media, (2) pediatrics, and (3) telehealth or digital health. The start date (January 2010) was selected because smartphone-enabled imaging and telehealth implementations relevant to pediatric otoscopy began to emerge and diffuse in clinical practice around that period. Full strategies for each database are provided exactly as run in Multimedia Appendix 1. The strategy was developed de novo and was not adapted from prior reviews, and no published search filters were used.

In addition, forward citation searching was performed in Web of Science (using “Times Cited” and “Cited Reference Search”) for key included articles, and reference lists of included studies were manually screened. Study registries were not searched, and study authors, experts, or manufacturers were not contacted for additional or missing information. The search strategy was not formally peer-reviewed using a dedicated tool but was iteratively reviewed within the author team. Duplicates were removed by automated matching of title, author, and publication year, followed by manual verification.

### Eligibility Criteria

We defined the eligibility criteria using the population, concept, and context (PCC) framework developed by the Joanna Briggs Institute (JBI):

- Population: children and adolescents aged <18 years with suspected or confirmed OM (AOM/OME/recurrent acute OM [RAOM]), including mixed-age studies if pediatric data were separable
- Concept: telehealth or digital health approaches for middle ear evaluation and/or OM management, including tele-otoscopy (image/video capture with remote review), tele-otoscopy plus adjunct testing (tympanometry, acoustic methods), and other clinically implemented digital tools relevant to OM assessment or follow-up
- Context: any clinical or community setting (primary care, ED, specialty clinics, schools, outreach programs, home)
- Types of sources: original research reporting clinical implementation outcomes (diagnostic, clinical, implementation, or economic outcomes)

We excluded conference abstracts, protocols, reviews or editorials, adult-only studies, and purely technical evaluations without clinical implementation.

### Data Items and Operational Definitions

We used “tele-otoscopy” to describe capture of otoscopic media (images/videos) for remote interpretation (store and forward or real time). We distinguished this from “video visits without otoscopy,” in which no ear imaging was obtained. “Adjunct digital diagnostics” referred to nonimaging tools to assess middle ear status (eg, tympanometry and smartphone acoustics).

### Study Inclusion Criteria and Exclusion Criteria

Studies were considered eligible if they were published in English between January 1, 2010, and February 9, 2026, and focused on telemedicine, telehealth, or other digital health interventions for pediatric OM. Eligible designs included qualitative, quantitative, or mixed-method approaches, provided that they reported outcomes of implementation in clinical practice. Studies that enrolled children and adolescents aged <18 years with suspected or confirmed OM—in which patients were assessed, monitored, or treated using telemedicine-supported interventions—were included.

Conference abstracts, reviews, editorials, and study protocols were excluded. Studies were also excluded if they focused exclusively on adult populations or conditions other than OM or those that did not involve the clinical application of telemedicine. Purely technical studies—including device prototype development and retrospective artificial intelligence model performance evaluations without prospective or clinical validation—and conference-only publications were excluded. Studies centered solely on audiology or hearing assessment without otoscopic or middle ear evaluation were also excluded.

### Selection of Sources of Evidence

Titles and abstracts were independently screened by 2 reviewers (MN and RA). Full-text articles were retrieved for studies deemed potentially eligible and assessed against the predefined inclusion and exclusion criteria. Studies were excluded at the full-text stage for reasons including lack of clinical implementation, exclusive focus on retrospective algorithm performance, conference-only publications, absence of pediatric populations, or absence of OM-specific outcomes. Any disagreements were resolved through discussion and consensus. As part of the revision process, the rerun search results were screened using the same procedures and criteria as the original search.

### Data Charting Process and Items

Data extraction was independently performed by 2 reviewers (MN and RA) using a standardized charting form. Extracted items included bibliographic details (author and year) and study design; country and setting (clinic, community, school, or home; level of care); population characteristics (age range, sample size, and OM phenotype when specified); care model (asynchronous, synchronous, or hybrid) and workflow description; operator roles (who captured and who interpreted) and training protocols (duration and content and any quality thresholds); technology and device or system characteristics (type, smartphone requirement, platform, and manufacturer when available); comparator and reference standard (otomicroscopy, pneumatic otoscopy, or surgical findings); outcomes (diagnostic performance, clinical outcomes, implementation outcomes, economic outcomes, and equity-relevant outcomes when reported); and study limitations, potential biases, and funding or conflict of interest statements when reported. Any discrepancies between reviewers were resolved through discussion and consensus. Some included studies reported diagnostic accuracy outcomes; thus, we conducted a targeted assessment for the subset of diagnostic accuracy studies using the revised Quality Assessment of Diagnostic Accuracy Studies (QUADAS-2) tool. Two reviewers independently assessed the risk of bias and applicability, with disagreements being resolved by consensus; detailed judgments are provided in Multimedia Appendix 2.

### Synthesis of Results

We summarized the extracted data descriptively (counts/percentages) and synthesized the findings narratively. We created evidence maps (technology category × care model; operator capture vs interpretation) and an outcome-domain frequency figure to visualize evidence distribution and gaps. To apply an implementation science lens, we mapped the reported implementation outcomes and determinants across included studies to the reach, effectiveness, adoption, implementation, and maintenance (RE-AIM) framework. A summary of the RE-AIM framework analysis of each study included in this review is provided in Multimedia Appendix 3.

## Results

### Selection of Sources of Evidence

**Figure 1.**
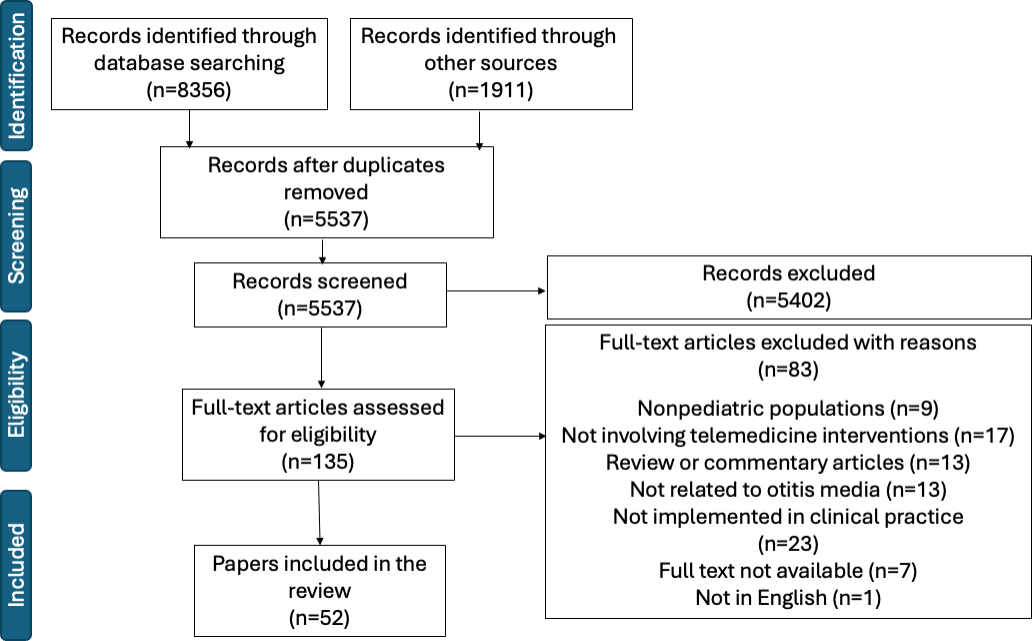
Preferred Reporting Items for Systematic Reviews and Meta-Analyses (PRISMA) flow diagram.

The database search retrieved 8356 articles from MEDLINE via PubMed (n=1687), Scopus (n=4810), Web of Science Core Collection (n=1775), and CENTRAL (n=84). In addition, 1911 records were identified through forward citation searching in Web of Science (and backward reference checking). Following the removal of duplicates, 5537 articles remained for the screening of titles and abstracts; of these, 5402 irrelevant articles were excluded, and 135 articles remained. The full texts of 135 articles were subsequently evaluated for eligibility; of these, 83 articles were excluded for the following reasons: nonpediatric population (n=9), absence of telemedicine interventions (n=17), review or commentary articles (n=13), unrelated to OM (n=13), not implemented in clinical practice (n=23), not in English (n=1), and unavailability of full text (n=7). Ultimately, 52 studies were included in this scoping review (Figure 1, PRISMA flow diagram).

### Characteristics of Sources of Evidence

The 52 included studies were published between 2012 and 2026, representing a geographically diverse evidence base encompassing high-, low-, and middle-income settings (summarized in Multimedia Appendix 2) [12-16,22-24,28-71]. QUADAS-2 judgements for diagnostic accuracy studies are summarized in Multimedia Appendix 4. The largest number of included studies (n=15) were conducted in the United States [39,41,42,44,49,52-55,58-60,66,67,71], followed by Australia (n=10) [14,22,23,28,31,32,61,64,65,70], the United Kingdom (n=5) [45,50,51,62,68], India (n=4) [35,40,46,57], and China (n=2) [16,56], with additional studies from other countries, including developing countries (Table 1).

**Table 1.** Geographic distribution of included pediatric otitis media telemedicine studies by country (N=52).

Country	Included studies, n (%)^a^	Author (year)
United States	15 (28.8%)	Rappaport et al (2016) [66], Richards et al (2015) [71], Shah et al (2018) [39], Chan et al (2019) [41], Chan et al (2019) [42], Hakimi et al (2019) [44], Jayawardena et al (2020) [49], Kolb et al (2021) [52], Kleinman et al (2021) [53], Don et al (2021) [54], Ni et al (2021) [67], Manayan et al (2022) [55], Schafer et al (2022) [58], Smola et al (2022) [59], Chan et al (2022) [60]
Australia	10 (19.2%)	Smith et al (2012) [28], Phillips et al (2014) [70], Smith et al (2015) [31], Jacups et al (2017) [32], Habib et al (2023) [61], Alenezi et al (2024) [14], Quick et al (2024) [22], Habib et al (2024) [23], Bowers et al (2025) [64], Altamimi et al (2025) [65]
United Kingdom	5 (9.6%)	Fordington and Holland Brown (2020) [45], Cottrell et al (2020) [50], Schuster-Bruce et al (2021) [51], Patel et al (2024) [62], Stancel-Lewis et al (2025) [68]
India	4 (7.7%)	Ramkumar et al (2018) [35], Bhavana et al (2018) [40], Gupta et al (2020) [46], Deshmukh et al (2022) [57]
China	2 (3.8%)	Meng et al (2022) [56], Jin and Fan (2024) [16]
France	2 (3.8%)	Venail et al (2018) [38], Dubois et al (2024) [15]
Nepal	2 (3.8%)	Mandavia et al (2018) [36], Gyawali et al (2024) [63]
South Africa	2 (3.8%)	Biagio et al (2014) [29], Lundberg et al (2017) [34]
Netherlands	1 (1.9%)	Prins-van Ginkel et al (2017) [33]
Brazil	1 (1.9%)	Wagner et al (2023) [13]
Canada	1 (1.9%)	Mousseau et al (2018) [37]
Finland	1 (1.9%)	Erkkola-Anttinen et al (2019) [12]
Greenland	1 (1.9%)	Demant et al (2019) [43]
Hungary	1 (1.9%)	Pannonhalmi et al (2025) [24]
Taiwan	1 (1.9%)	Wu et al (2014) [30]
Turkey	1 (1.9%)	Durgut et al (2020) [48]
Republic of Korea	1 (1.9%)	Cha et al (2020) [47]
Kenya	1 (1.9%)	Yancey et al (2019) [69]

a Percentages may not sum to 100% due to rounding.

Designs encompassed diagnostic accuracy comparisons, pragmatic service evaluations, prospective and retrospective cohorts, case series, and education and simulation trials; most studies either enrolled children exclusively or included defined pediatric subsets. Across studies, sample sizes ranged from small pilot cohorts to large-scale community and school-based screening programs.

### Results of Individual Sources of Evidence

#### Telemedicine Models, Users, and Settings

Across the included studies, asynchronous store-and-forward approach was the most commonly used approach, accounting for 35 studies (67.3%; Table 2).

**Table 2.** Telemedicine care model classification across included studies on pediatric otitis media (N=52).

Telemedicine model	Included studies, n (%)^a^	Author (year)
Asynchronous^b^	35 (67.3%)	Smith et al (2012) [28], Biagio et al (2014) [29], Phillips et al (2014) [70], Smith et al (2015) [31], Rappaport et al (2016) [66], Prins-van Ginkel et al (2017) [33], Lundberg et al (2017) [34], Mandavia et al (2018) [36], Shah et al (2018) [39], Bhavana et al (2018) [40], Chan et al (2019) [42], Demant et al (2019) [43], Erkkola-Anttinen et al (2019) [12], Yancey et al (2019) [69], Fordington and Holland Brown (2020) [45], Gupta et al (2020) [46], Cha et al (2020) [47], Durgut et al (2020) [48], Cottrell et al (2020) [50], Don et al (2021) [54], Ni et al (2021) [67], Manayan et al (2022) [55], Meng et al (2022) [56], Deshmukh et al (2022) [57], Smola et al (2022) [59], Chan et al (2022) [60], Habib et al (2023) [61], Alenezi et al (2024) [14], Patel et al (2024) [62], Dubois et al (2024) [15], Jin and Fan (2024) [16], Habib et al (2024) [23], Gyawali et al (2024) [63], Altamimi et al (2025) [65], Pannonhalmi et al (2025) [24]
Hybrid^c^	6 (11.5%)	Ramkumar et al (2018) [35], Jayawardena et al (2020) [49], Wagner et al (2023) [13],Quick et al (2024) [22], Bowers et al (2025) [64], Stancel-Lewis et al (2025) [68]
Synchronous^d^	11 (21.2%)	Wu et al (2014) [30], Richards et al (2015) [71], Jacups et al (2017) [32], Mousseau, et al (2018) [37], Venail et al (2018) [38], Chan et al (2019) [41], Hakimi et al (2019) [44], Schuster-Bruce et al (2021) [51], Kolb et al (2021) [52], Kleinman et al (2021) [53], Schafer et al (2022) [58]

a Percentages may not total 100% because of rounding.

b Asynchronous: capture of images or videos or data with later remote interpretation.

c Hybrid: combination of stored media and real-time guidance or interconsultation within the care process.

d Synchronous: real-time assessment or interaction (typically video visit or live otoscopy visualization).

In this approach, images or videos were captured by nonphysicians or caregivers and interpreted remotely by otolaryngologists. This includes laptop-tethered video-otoscopy in primary care [29], smartphone otoscopy with consultant review in rural clinics [36], parent-submitted videos in specialty care [39], nurse or technician capture with specialist interpretation in community programs [61], and a multidisciplinary pathway using scheduled remote reviews [14].

Synchronous models accounted for 11 of 52 studies (21.2%) and included real-time postoperative reviews and acute assessments [32,37,52,53,58], whereas blended stored media with live guidance or interconsultation accounted for 6 of 52 studies (11.5%) [13,22,35,49,64,68]. Telemedicine-supported otologic care was implemented across a variety of settings, including primary care clinics, emergency departments, specialty outpatient services, schools, community screening programs, and home environments.

#### Technologies and Devices

A diverse range of hardware and software solutions were reported across the included studies (Table 3).

**Table 3.** Summary of technologies and key outcomes across included pediatric otitis media telemedicine studies (N=52).

References	Implementation/clinical outcome	Diagnostic performance	Typical setting	Representative device/system	Technology category
[12,36,37,41,44,49-51,53,54,71]	Repeat confirmatory examinations decreased from 97.9% to 27.2% when digital otoscopy was used in training clinics [53]. Image acquisition required a median of ~6 minutes per case in rural outpatient workflows [36]. For home tympanostomy tube surveillance, clinicians completed remote reviews in 1‐3 minutes, families reported mean savings of ~US $127 per episode, and satisfaction was high [54].	Diagnostic concordance with in-person otoscopy was 95%, with κ=0.89, sensitivity 0.94, and specificity 0.96 [36]. In a randomized comparison, trainee diagnostic accuracy improved by 11.2% using digital otoscopy, and interrater agreement rose to Fleiss κ^a^=0.69 versus Fleiss κ=0.40 with a conventional otoscope [53]. Parent-captured videos yielded 40% diagnostic-quality submissions, allowed 87% detection or exclusion of AOM^b^ during acute episodes, and achieved κ=0.69 between raters [12].	Emergency, outpatient, school, home; clinicians, trainees, parents	CellScope Oto, Cupris TYM, TYMPA system, endoscope-i	Smartphone-connected digital otoscopes or otoendoscopes
[29,32,34]	Short, focused training improved diagnostic agreement and enabled reliable store-and-forward workflows in primary care [29,34].	Agreement with otomicroscopy was Cohen κ=0.68‐0.75, with 72.4%‐79.3% sensitivity and 93.2%‐98% specificity [29]. In facilitator-collected video-otoscopy reviewed by a general practitioner, repeat video review improved weighted κ from 0.69 to 0.82 (OMgrade), and specificity reached 0.98 [34].	Primary care; CHWs^c^ or GPs^d^	Dino-Lite Pro Earscope	USB video-otoscopes
[42]	Users constructed the funnel in a mean (SD) time of 2.8 (0.9) minutes and rated usability as 8.9/10; performance was consistent across phone models following calibration.	Discrimination of middle ear effusion achieved an AUC^e^ 0.898, with 84.6% sensitivity and 81.9% specificity, outperforming commercial acoustic reflectometry (AUC 0.776). Caregivers’ classifications closely matched that of clinicians in 24/25 ears [42].	Home; caregivers	Paper funnel + smartphone microphone	Smartphone-based acoustic system
[60]	Materials cost was <US $30 while maintaining functional parity with a commercial device; the clinical cohort excluded infants younger than 9 months, defining the initial applicability window.	Classification by 5 audiologists, 86% (2%) agreement; all Type B correctly identified. Bland-Altman in 45 intact ears: peak admittance bias=–0.02 (0.14) mL, peak pressure bias=–1 (17) daPa, and ear canal volume bias=–0.09 (0.25) mL [60].	Outpatient; audiologists	Open-source prototype vs GSI TympStar	Low-cost smartphone tympanometer
[33,45,70]	Adoption was high, with 87% of families downloading the app and 74% using it between visits, and clinicians reported improved information exchange during care [45].	A clinic-based app hearing test correlated with pure-tone audiometry (r=–0.656) [45]. In a cohort comparison, the app arm recorded a higher proportion of symptom days (44%) than the paper diary arm (32.5%) and achieved near-complete questionnaire return [33].	Home; parents/patients	Diary tracking, Hear Glue Ear, Mimi	mHealth applications
[15,16,47]	Systems supported self-examination and automated triage, yet generalizability across sites, devices, and pediatric populations remains limited pending broader external validation.	Internal validation of a CNN^f^ model reported sensitivity and specificity >98% with AUROC^g^ 1.00 across classes; external testing showed reduced sensitivity for AOM (58.3%) and OME (58.3%) with high specificity (100% and 98.1%) [15]. A low-cost USB device with DenseNet^h^ achieved 95.7% overall accuracy; for OME^i^, sensitivity 0.77 and specificity 0.99; for cholesteatoma, sensitivity 0.79 [16]. A rule-based model reached Top-1 accuracy of 69.5% versus 83% by human telemedicine reviewers [47].	Mixed otology; clinicians and self-examination users	Karl Storz Smart Scope + i-Nside (Inception-v2), DenseNet USB endoscope, rule-based Android	Artificial intelligence–assisted digital otoscopy
[13,22,64]	Image capture success reached 96.3% during remote sessions [22]. School programs achieved >85% coverage and reduced costs relative to standard pathways and were rated highly by families, supporting feasibility for integrated tele-audiology [13,64].	In school-entry children, remote testing matched face-to-face within ±10 dB for air conduction at 1 kHz (98%) and 4 kHz (97.8%), bone conduction matched in 100%/95.7%, otoscopy agreement was κ=0.60, and tympanometry agreement was κ=0.90 [64]. Live video-otoscopy showed substantial agreement (κ=0.68 for videos; κ=0.83 for still images) and identified more landmarks (2.79 vs 1.68) [22]. Using a multisensor kit in pediatrics, ear-specific sensitivity was 64.4% (tympanic membrane) and 66.1% (ear canal), with specificity consistently above 70% [13].	School and pediatric tele-audiology	TytoPro; Otometrics A450 + Aurical Otocam 300 + Interacoustics MT10	Multisensor or hybrid diagnostic kits
[14,65]	Median time to diagnosis and care plan fell to 28 days compared with 450 days in standard care (*P*<.001) [14]. A hospital cost analysis estimated ~US $67 per initial visit (vs US $155) with a break-even point at 223 appointments and about 3-fold greater specialist throughput [65].	Performance was driven by the integrated, multitest pathway rather than a single diagnostic metric [14,65].	Multidisciplinary teleclinic	“Ear Portal”: HearScope + HearScreen + Titan (WBT^j^) + DPOAE^k^	Integrated multidisciplinary pathway
[28,31,43,46,57,69]	In a 45-camp program, 3000 individuals were screened; 54% were referred, 13% presented to hospital, and roughly half of presenters required surgery; procedures were delivered at about 40% of usual cost [46]. Longitudinal regional programs achieved ~85% coverage and reduced referrals and waits [28,31].	In CHW-led screening, overall sensitivity was 96.9% with condition-specific concordance of CSOM^l^ 96.2%, wax 98%, ASOM^m^ 93.9%, foreign body 100%, and OME 100% [57]. When operators received only written instructions, diagnostically useful video yield was 18.1% with substantial interrater agreement (Fleiss κ=0.67) and higher usefulness in older children [43].	Community/schools; CHWs, teachers	ENTraview + ClickMedix; Mebird T5 video-otoscopy + EMRs^n^ support follow-up	Community and school screening programs
[48,55]	High false-positive rates led to a 66% referral rate in a cohort with 27% hearing loss prevalence; performance was limited by ambient noise and lack of standardized calibration [55].	The Hearing Test app showed κ=0.059, indicating poor agreement with conventional audiometry [48]. The hearScreen app achieved 85% sensitivity and 41% specificity, with ambient noise at 500 Hz significantly increasing false positives (*P*=.01) [55].	Clinics/non-soundproof rooms	Hearing Test, hearScreen	App-based hearing screening

a κ: Cohen kappa.

b AOM: acute otitis media.

c CHW: community health worker.

d GP: general practitioner.

e AUC: area under the receiver operating characteristic curve.

f CNN: convolutional neural network.

g AUROC: area under the receiver operating characteristic curve.

h DenseNet: Dense Convolutional Network.

i OME: otitis media with effusion.

j WBT: wideband tympanometry.

k DPOAE: distortion product otoacoustic emissions.

l CSOM: chronic suppurative otitis media.

m ASOM: acute suppurative otitis media.

n EMR: electronic medical record.

The most frequently used solution was smartphone-connected digital otoscopes and otoendoscopes, such as CellScope Oto, Cupris TYM, TYMPA system (TympaHealth), and endoscope-i, which were applied in emergency, outpatient, school, and home settings [11,36,37,41,44,49-51,53,54,71]. Earlier clinical evaluation of smartphone-enabled otoscopy for OM diagnosis and management were reported, supporting feasibility in routine care [66]. USB video-otoscopes, including the Dino-Lite Pro Earscope, were used to support store-and-forward apps in clinics [29,34]. Non-smartphone digital otoscopes, such as Welch Allyn Digital MacroView Otoscope, TeleHealth Flexican Otoscope, and Welch Allyn USB Otoscope, were used for real-time follow-up and teleconsultations [22,32].

Apart from imaging, several digital tools were used. A smartphone-only acoustic system, using a paper funnel and the phone’s microphone, enabled the detection of middle ear effusion (MEE) [42]. Mobile health (mHealth) apps facilitated diary-based episode detection and home management [33,45]. By contrast, although acceptability was high, a culturally tailored multimedia or text messaging intervention for Aboriginal children with tympanic membrane perforation did not improve clinic attendance or short-term ear health outcomes [70]. A low-cost, open-source smartphone tympanometer was evaluated against the GSI TympStar systems (Grason-Stadler) [60]. Decision support innovations included the Karl Storz Smart Scope with the i-Nside app (Inception-v2) and an inexpensive (~US $10) USB endoscope integrated with a Dense Convolutional Network (DenseNet) classifier for self-examination [15,16]. A consumer Android-tethered otoendoscope, coupled with a rule-based model, demonstrated performance comparable to that of clinicians in diverse otology settings [47]. Community programs frequently used Medtronic’s ENTraview integrated with ClickMedix, and 1 program used the Mebird T5 SO (Black Bee Intelligent Manufacturing [Shenzhen] Technology Co., Ltd.) for asynchronous case discussion [35,46,56,57]. A multisensor kit (TytoPro) enabled remote pediatric interconsultation [13]. The school-based hybrid audiology program integrated Madsen A450 audiometry (Otometrics Natus Medical), Natus Aurical Otocam 300 video-otoscopy, and Interacoustics MT10 tympanometry with videoconferencing [64]. For training, Otosim2 (OtoSim Inc.) via AudioProConnect and a low-cost KZYEE USB otoscope improved teaching and landmark recognition [38,44]. Teslong pen endoscopes and the Mimi Hearing Test app (Mimi Hearing Technologies) were piloted for supervised home capture and hearing checks [49,63]. A structured “Ear Portal” pathway combined hearX HearScope video-otoscopy, HearScreen audiometry, Interacoustics Titan wideband tympanometry, and distortion product otoacoustic emissions into a scheduled multidisciplinary review [14,65].

#### Technology Category × Care Model

**Figure 2.**
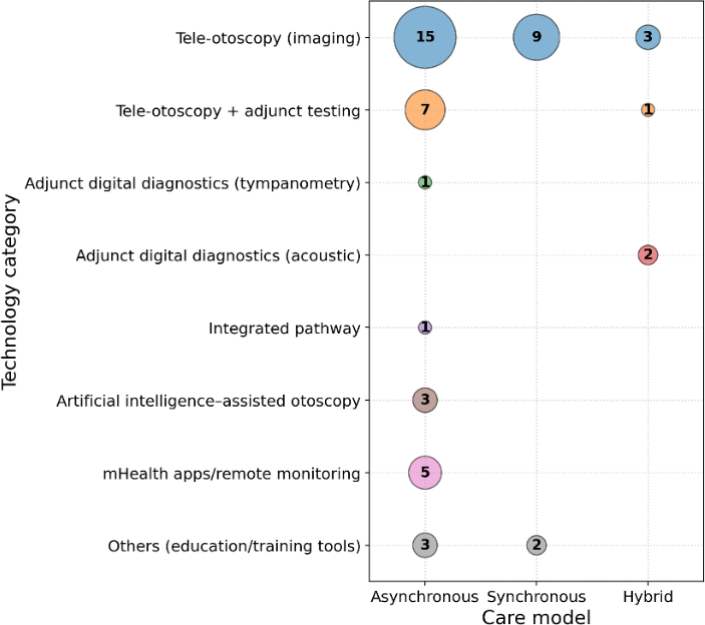
Evidence gap map of technology category by care model in pediatric otitis media telemedicine. mHealth: mobile health.

Figure 2 maps technology categories against care models. Most studies used asynchronous store-and-forward workflows, primarily tele-otoscopy imaging alone or tele-otoscopy combined with adjunct testing (Figure 2). Hybrid models were less common and tended to involve multisensor kits or tele-otoscopy plus adjunct testing. Notable gaps included limited evidence for integrated pathway models beyond a small number of programs and sparse evaluation of outcomes beyond diagnostic performance, including long-term child outcomes and cost-effectiveness.

#### Operators and Workflow Roles

**Figure 3.**
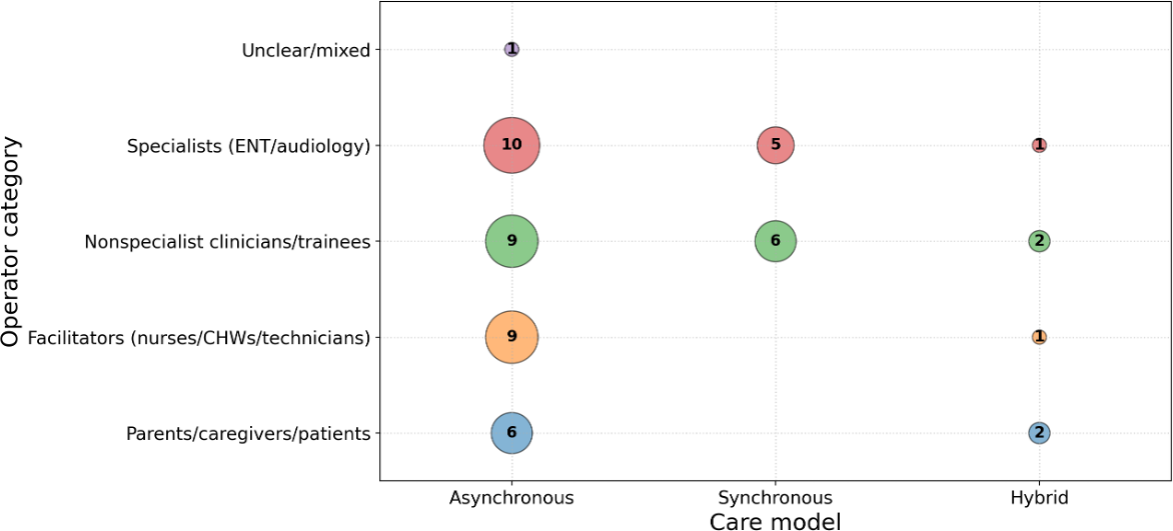
Operator workflow map showing who captures otoscopic media and who interprets it in pediatric otitis media tele-otoscopy. CHW: community health worker, ENT: ear, nose, and throat.

Figure 3 summarizes who captured otoscopic media (clinicians, nurses/community facilitators, caregivers/parents) and who interpreted it. Studies consistently indicated that diagnostic yield depended strongly on operator training and workflow support, which includes checklists, feedback loops, and cerumen management. Evidence gaps included limited direct comparisons of training intensity and limited reporting of minimum image quality thresholds.

### Diagnostic Performance

Across diagnostic accuracy studies, the reported κ, sensitivity, and specificity varied by key sources of clinical and methodological heterogeneity, including pediatric age strata, OM phenotype definitions (AOM vs OME vs mixed), reference standards, operator training and experience, and device and workflow-specific image quality constraints (Table 4). To improve interpretability, we summarized diagnostic performance by capture-interpretation model and reference standard.

**Table 4.** Key sources of clinical and methodological heterogeneity across diagnostic accuracy studies in pediatric otitis media tele-otoscopy.

Study and category	Key heterogeneity						Performance (κ^a^/Se^b^/Sp^c^/AUC^d^)
	Age	Spectrum	Operator/training	Reference standard	Device	Quality	
(A) Trained operator/expert interpretation							
Biagio et al (2014) [29]	2-16 y	Normal/abnormal OM	Trained capture → clinician read (2 sessions)	Otomicroscopy	Asynchronous video-otoscopy	Acceptable/excellent ratings (NR criteria)	κ 0.68‐0.75; Se 72.4%‐79.3%; Sp 93.2%‐98%
Lundberg et al (2017) [34]	Pediatrics (NR)^e^	Normal/OME^f^/AOM^g^/CSOM^h^ (abnormal pooled)	Facilitator capture → GP^i^ read (repeat)	Otologist otomicroscopy	Video-otoscopy vs otoscopy	NPD^j^ due to wax/degree of cooperation/low quality	wκ^k^ 0.76→0.82 (video) vs 0.69 (otoscopy); Se 0.77‐0.81; Sp 0.94‐0.98
Mandavia et al (2018) [36]	Pediatrics (NR)	Screening (NR)	Trainees capture → remote consultant read	On-site specialist otoscopy/referral	Smartphone otoscopy (Cupris TYM)	NR	Concordance 95% (κ=0.89); Se 0.94; Sp 0.96; referral concordance 100%
(B) Parent/nonspecialist capture (training dependent)							
Erkkola-Anttinen et al (2019) [12]	6-35 mo	AOM-focused	Parent capture (hands-on teaching) → remote raters	Clinician assessment (NR)	Device: smartphone video-otoscopy	Sufficient quality 62%‐64% during intervention; diagnostic quality 40%	Detect/exclude AOM κ=0.69 (87% possible)
Shah et al (2018) [39]	3 mo to 17 y	Mixed pathology	Parent or physician capture → physician read (brief tutorial)	Pneumatic otoscopy (± tympanometry subset)	iPhone/CellScope	Wax common; wax-alert κ≈0.01	Parent κ=0.42; physician κ=0.74 (vs pneumatic otoscopy)
Demant et al (2019) [43]	6-72 mo	Usefulness outcome	Nonspecialist HW capture (written instructions only) → ENT^l^ raters	NR	Smartphone otoscopy (Cupris TYM)	Useful videos 6.6% (<24 mo), 22.7% (24-48 mo), 30.7% (>48 mo)	Modified Fleiss κ 0.73 (<24 mo), 0.48 (24-48 mo), 0.60 (>48 mo)
(C) Different outcomes/surrogate standards/different modalities							
Kleinman et al (2021) [53]	Pediatrics (NR)	Education/clinical trial	Trainees	Education/supervisor (NR gold standard)	Digital vs traditional otoscope	NR	Accuracy +11.2%; Fleiss κ 0.40→0.69; repeat confirmatory examinations 97.9%→27.2%
Rappaport et al (2016) [66]	6 mo to 18 y	AOM via ED^m^ diagnosis (ICD-9^n^ surrogate)	ED capture → blinded physician reviewers	ED diagnosis	Smartphone vs digital otoscope	NR	Overall κ=0.375; smartphone κ=0.368; digital κ=0.381
Chan et al (2019) [42]	18 mo to 17 y (median 5 y)	Chronic OME/recurrent AOM vs controls	Clinician testing;parental use tested after brief demo	Surgical fluid (myringotomy) and/or ENT pneumatic otoscopy	Smartphone acoustic method (paper funnel; ML)^o^	Phone model calibration	AUC 0.898; Se 84.6%; Sp 81.9%; parents matched clinician 24/25 ears
Durgut et al (2020) [48]	5-15 y	OME (50 children; 88 ears with OME + 12 controls)	In-clinic testing	Booth pure-tone audiometry	Android Hearing Test app	Environmental noise limitation	Very poor agreement: κ=0.059 (100 ears); no significant correlation 500‐4000 Hz

a κ: Cohen kappa.

b Se: sensitivity.

c Sp: specificity.

d AUC: area under the receiver operating characteristic curve.

e NR: not reported.

f OME: otitis media with effusion.

g AOM: acute otitis media.

h CSOM: chronic suppurative otitis media.

i GP: general practitioner.

j NPD: not possible to diagnose.

k wκ: weighted kappa.

l ENT: ear, nose, and throat.

m ED: emergency department.

n ICD-9: International Classification of Diseases, 9th Revision.

o ML: machine learning.

#### Trained Operator Capture with Expert Interpretation Against Clinical Reference Standards

When images were captured by trained operators and interpreted by experienced clinicians, store-and-forward tele-otoscopy showed substantial agreement with in-person otomicroscopy. In a primary care cohort, asynchronous video-otoscopy yielded Cohen κ values of 0.68‐0.75 across raters and 2 review sessions, 72.4%‐79.3% and specificities of 93.2%‐98% [29]. In another clinic, repeated readings improved weighted κ from 0.69 to 0.82, with specificity up to 0.98 [34]. Smartphone otoscopy used by trainees, with remote consultant review, achieved 95% ear-level concordance with on-site specialist otoscopy (κ=0.89; sensitivity 0.94; specificity 0.96) and 100% concordance with referral decisions [36]. For training, pediatric emergency department residents demonstrated similar diagnostic accuracy when using a smartphone device compared with a traditional otoscope (0.74 vs 0.69; nonsignificant). However, novice users tended to favor the conventional tools, as they felt confident with them [37]. A separate academic comparison showed that digital otoscopy improved trainee accuracy by 11.2%, increased interrater agreement (from Fleiss κof 0.40 to 0.69), and reduced repeat confirmatory examinations from 97.9% to 27.2% [53].

#### Parent and Nonspecialist Capture (Training- and Cooperation-Dependent Performance)

Parent and nonspecialist performance was highly dependent on training and cooperation. After structured teaching, parents produced videos of sufficient quality in 62%‐64% of submissions during the intervention periods, and the evaluators showed substantial agreement for detecting or excluding AOM (κ=0.69) [12]. In contrast, a brief tutorial yielded lower agreement with pneumatic otoscopy (parent κ=0.42 vs physician κ=0.74), while automated cerumen alerts showed negligible agreement (κ=0.01), highlighting the advantage of structured teaching [39]. Without hands-on training, local health care workers produced diagnostically useful videos in only 18% of cases, with insertion errors and cerumen as major barriers; usefulness increased proportionally with child age [43]. Educational approaches also included a smartphone-adaptable video-otoscopy quiz developed to support otoscopic image interpretation skills, reflecting ongoing efforts to standardize competency development for tele-otoscopy workflows [67].

#### Alternative Modalities and/or Surrogate Outcomes (Interpret with Caution)

Beyond optical otoscopy workflows, a smartphone-based acoustic method to detect MEE achieved an area under the receiver operating characteristic curve (AUC) of 0.898, with approximately 85% sensitivity and 82% specificity under surgical and/or specialist reference standards, and caregiver performance approximated clinician performance in a subset [42]. A smartphone tympanometer also demonstrated approximately 86% classification agreement with commercial devices, with only minor bias in peak admittance and pressure, supporting the feasibility of adjunct middle ear assessments beyond optical imaging [60]. In contrast, a pediatric hearing test app demonstrated very poor agreement with booth audiometry (κ≈0.06) [48,55]. These findings underscore that performance estimates are not directly comparable across studies when index test modality, outcome definition, and reference standards differ. Artificial intelligence–supported classifiers have shown high performance on curated datasets but lower sensitivity for OM and limited external validation across devices and sites [15,16,47].

### Clinical and Implementation Outcomes

#### Comparison With Traditional Approaches

Tele-enabled programs have broadened access and accelerated clinical decision-making. A school or community program completed 2111 assessments in 1053 children, demonstrating 85% coverage. In another evaluation, it reduced waiting times and tertiary referrals as the program expanded [28,31]. A general practitioner-to-ear, nose, and throat (ENT) specialist smartphone endoscopy pathway was able to manage 83% of the 53 referrals remotely, with a median specialist response time of 48 (IQR 22-148) minutes [50]. In a pediatric emergency department, a smartphone otoscope showed substantial within-physician agreement versus a traditional otoscope and changed the reported tympanic membrane view in 12%‐16% of examinations, including clinically relevant diagnostic changes to or from AOM in 6%‐7% of examinations [71]. Community screening of 3000 individuals generated 54% of referrals, but only 13% were ultimately presented for hospital evaluation; nearly half of those evaluated required surgery, underscoring the barriers of pathway follow-through [46]. A telehealth-supported surgical model achieved 80% clinical resolution, 88% hearing improvement, and reduced travel costs for postoperative review [32]. Home tympanostomy tube surveillance demonstrated high interrater and intrarater agreement, rapid clinician review (1‐3 min), and high family satisfaction with reported time and cost savings [54]. The Ear Portal pathway substantially shortened time to diagnosis and care plan (median 28 [IQR 19.8] days), and an economic analysis showed that it lowered per-visit costs, reached a break-even point after ~223 appointments, and increased specialist throughput [14,65]. A community service integrating smartphone otoscopy with tablet audiometry discharged most patients at their first visit at a lower cost than other services using conventional methods, with specialist review rarely leading to changed decisions [62]. However, televisits, which were performed predominantly without ear imaging, produced similar rates of tube recommendations but substantially lower documentation of effusion at surgery compared with encounters that incorporated ear imaging, indicating a potential risk of suboptimal decision-making when otoscopy is omitted [52]. During the COVID-19 pandemic, telehealth encounters were overall less likely to end with tube recommendations, underscoring the limitations imposed by restricted physical examination [58]. A multisensor interconsultation device demonstrated high agreement for several systems but lower sensitivity for ear canal and tympanic membrane findings than conventional methods, reinforcing the necessity of dedicated otoscopy using generalist kits [13]. In pediatric telehealth, live video-otoscopy provided superior image quality and landmark visualization compared to still images, supporting synchronous visualization when feasible [22]. A school-entry hybrid model demonstrated close agreement with face-to-face audiometry and tympanometry, with acceptable agreement for otoscopy [64].

#### Outcome Domains Reported

Table 5 summarizes the distribution of outcome domains reported across included studies (N=52). Diagnostic performance was the most frequently reported domain (38/52, 73.1%), followed by implementation and usability outcomes (29/52, 55.8%). Clinical and management impact (22/52, 42.3%) and audiology and tympanometry outcomes (19/52, 36.5%) were reported less often, while image or capture quality was reported in 15 of 52 studies (28.8%). Training and education and economic outcomes were each reported in 8 of 52 studies (15.4%), and artificial intelligence or automation was reported in 4 studies (7.7%; Table 5). Reports of longer-term patient outcomes and adverse events were uncommon, indicating priorities for future research.

**Table 5.** Outcome domains reported across included studies (N=52).

Outcome domain	Studies reporting domain, n (%)^a^
Diagnostic performance	38 (73.1%)
Implementation/usability	29 (55.8%)
Clinical/management impact	22 (42.3%)
Audiology/tympanometry outcomes	19 (36.5%)
Image/capture quality	15 (28.8%)
Training/education	8 (15.4%)
Economic outcomes	8 (15.4%)
Artificial intelligence/automation	4 (7.7%)

a Studies may contribute to multiple domains; therefore, percentages do not sum to 100%.

#### Applications and Implementation Feasibility

Tele-enabled tools were applied in 4 management scenarios for pediatric ear diseases. For AOM diagnosis, asynchronous video-otoscopy demonstrated substantial agreement with otomicroscopy when images were captured by trained operators, as reported in South Africa using a laptop-tethered Dino-Lite Pro Earscope and specialist review [29] and in a primary care study in which repeated readings of stored video sequences closely tracked the microscopic reference [34]. At the bedside, smartphone-based digital otoscopy (CellScope Oto) demonstrated accuracy comparable to, or modestly better than, that of trainees using a conventional otoscope in a pediatric ED [37]. With structured instructions, parents successfully captured diagnostically useful otoscopy videos at home, supporting remote triage for suspected AOM [12]. For posttympanostomy tube surveillance and postoperative follow-up, home digital otoscopy with CellScope Oto enabled asynchronous specialist review and was rated highly acceptable by families. In remote Australian communities, a telehealth-supported surgical pathway cut travel and costs for postoperative care [32,54]. For screening and triage in underserved settings, school- and community-based programs—ranging from mobile ear-screening services in Queensland to large-scale campaigns using the ENTraview smartphone otoscope integrated with the ClickMedix platform—efficiently detected pathology, shortened time to specialist input, and improved surgical case prioritization, although barriers such as transport and out-of-pocket costs limited follow-through [28,46]. Additionally, a recent parent-led home ear health check pilot targeting children with complex needs highlighted feasibility and potential access benefits for populations facing barriers to clinic-based otoscopy [68].

Although feasibility was consistently high across settings and devices, the diagnostic yield depended on operator training, child cooperation, and cerumen management. Parents performed better after structured teaching, but accuracy declined with only brief instructions or when wax removal was not addressed [39]. Hands-on practice with a live tele-otoscopy simulation using Otosim2 with the AudioProConnect platform preserved accuracy and shortened task time, whereas written-only instructions under field conditions produced a low proportion of diagnostically useful videos [43]. Clinicians generally rated smartphone otoscopy imagery as adequate and highlighted supervisory advantages of shared visualization, including fewer repeat confirmatory examinations during training encounters [41,53]. Conversely, environmental noise and calibration issues materially influenced app-based hearing tools, which demonstrated poor agreement with booth-based audiometry in children with OME, and a smartphone screening app demonstrated high sensitivity but low specificity when ambient noise was not well controlled [48,55].

#### Risk of Bias and Study Limitations

Limitations include small, single-site samples; convenience sampling; mixed adult and pediatric cohorts in some studies; lack of blinded reference standards; heterogeneous operator training; device or discontinuation issues; and industrial involvement in some implementations. Parent and nonspecialist performance was sensitive to training and cerumen management, while omitting otoscopy in televisits risked misclassification of MEE [52].

#### Key Practical Takeaways

Across the included studies, the following points were notably identified as key practical takeaways:

1. Diagnostic performance depended strongly on who captured the otoscopic media and on the training and quality protocol rather than on the device alone.

2. Predefined image quality thresholds, multiview capture, and repeat capture when needed materially affected interpretability and downstream clinical decisions.

3. Telehealth encounters conducted without otoscopic imaging risk underdetecting middle ear pathology, including effusion, and may therefore alter management decisions when objective findings are required.

4. Hub-based or hybrid workflows—such as facilitated capture with remote review—appear to support access and feasibility while maintaining diagnostic reliability in many settings.

5. Evidence remains limited regarding scale-up outcomes (including sustainability/maintenance), formal economic evaluations (particularly in lower-middle-income countries), and external validation of artificial intelligence–enabled tools across devices and sites.

## Discussion

### Principal Findings

This scoping review mapped a rapidly evolving evidence base for telehealth in pediatric OM. Three consistent signals were observed. First, when images were captured using standardized techniques by trained operators and interpreted by experienced clinicians, store-and-forward tele-otoscopy demonstrated diagnostic performance similar to in-person examination. In primary care and community clinics, agreement with otomicroscopy was frequently in the substantial range, with high specificity for excluding the disease [29,34,36]. Second, operator training proved pivotal: performance declined markedly with brief, unsupervised capture by parents or lay workers; agreement with pneumatic otoscopy was low; and fewer than 20% of videos were diagnostically useful [43]. However, structured instruction or simulation improved the performance of workers without hands-on training [38,39,43]. Example of practical structured training can remain minimal, such as a 5- to 10-minute tutorial, a 1-page capture checklist, and brief hands-on coaching including cerumen management. Embedding a simple feedback loop on image quality further improves yield without adding substantial workload. Third, adjunct digital tools are promising: a smartphone-only acoustic method that infers tympanic membrane mobility detected MEE with the AUC approaching 0.90, whereas digital otoscopy platforms improved trainee-supervisor agreement and reduced duplicate examinations [42,53]. The contribution of this review is an implementation-facing synthesis that links “which tool,” “which care model,” and “under what conditions” to observed diagnostic performance, thereby informing scalable hybrid workflows.

### Interpretation and Implications

#### Diagnostic Performance and Care Models

When image acquisition was standardized and performed by trained facilitators, remote specialists and generalists demonstrated diagnostic agreement with in-person reference standards that are clinically feasible for triage and management in many settings [29,34,36,50]. Programs embedding store-and-forward video-otoscopy, tympanometry, and audiometry in school- and community-based screening have demonstrated high coverage, efficient specialist sorting, reduced wait times, and identified surgical candidates [28,31,64]. In primary care, asynchronous tele-referral pathways allowed most cases to be managed without face-to-face review while offering timely specialist input and educational feedback to referrers [50].

Simultaneously, telehealth visits performed without otoscopic visualization carry a risk of misclassification. In a pandemic-era cohort, children evaluated for RAOM via video-only visits were less likely to have effusion confirmed at the time of tympanostomy tube placement than office-based comparators (39% vs 79%), highlighting that history alone is insufficient for surgical decision-making [52]. These findings support hybrid models—telehistory plus rapid access to digital otoscopy (clinic kiosk, drive-through nurse station, and community facilitators) or validated home solutions—especially when management depends on objective middle ear findings [52,54,68].

#### Operators, Training, and Human Factors

Evidence has consistently demonstrated that the person capturing the image is as critical as the device used. Across included studies, capture quality and diagnostic yield varied substantially by operator type, training intensity, child cooperation, and cerumen burden [12,39,43]. Hands-on coaching and structured capture approaches were associated with higher proportions of interpretable recordings in parents and nonspecialists, whereas written-only or brief instruction yielded lower rates of diagnostically useful media in field settings [12,39,43]. In simulation settings, trained facilitators using Otosim2 with the AudioProConnect platform maintained expert accuracy without time penalties [38]. In clinical supervision settings, digital otoscopy improved trainee accuracy by approximately 11 percentage points and increased trainee-supervisor agreement from kappa 0.40 to 0.69, while reducing confirmatory reexaminations from ~98% to ~27% [53]. A randomized comparison in pediatric emergency department demonstrated similar accuracy for smartphones versus conventional otoscopy (0.74 vs 0.69) but revealed lower confidence among inexperienced users, emphasizing the value of familiarization [37]. Smartphone otoscopy may meaningfully affect clinical impressions. In a pediatric emergency department study, CellScope Oto altered the reported view in 12% to 16% of exams, with clinically relevant diagnostic changes in approximately 6%; notably, it was rated by clinicians as easy to use and valuable for teaching [71].

Given the established effectiveness of video-otoscopy, careful consideration of the methods and timing of use may enable promising integration [72]. Standardized training packages and minimum quality thresholds should be considered prerequisites for scaling, particularly for parents, community health workers, and very young children, in whom cooperation and ear canal size may complicate capture [12,39,43].

#### Adjunct Technologies and Artificial Intelligence–Based Decision Support

Smartphone-only acoustic reflectometry with embedded machine learning demonstrated high accuracy for effusion detection and performed comparably across platforms and even in parents’ hands [42]. This suggests a low-barrier screening pathway that does not depend on optical access, which is particularly valuable in toddlers with narrow ear canals or when video quality is suboptimal [42,43]. Early studies of automated image classification demonstrated throughput advantages, but clinical readiness remains limited due to scarce external validation across devices, sites, and pediatric populations and incomplete reporting of training datasets and labeling methods, which constrains generalizability and risk-of-bias assessment [15,16,47]. Although numerous models were developed using artificial intelligence to classify middle ear diseases on the basis of tympanic membrane findings or short-wave infrared imaging, there were few studies addressing its actual clinical implementation [15,16,73-76]. It is therefore necessary to consider not only the accuracy of these models but also the optimal point and method of integration within the clinical workflow [15,47]. Most studies also provide limited information on deployment pathways, including regulatory considerations, governance for performance monitoring, and safeguards when image quality is insufficient, and direct comparisons with human experts under identical capture conditions remain uncommon [15,47]. Accordingly, priority next steps include multisite, device-agnostic external validation using real-world pediatric data and prospective evaluations of workflow-level impact and safety rather than reliance on curated test-set metrics alone. In parallel, self-management apps for OME demonstrated high acceptability and reasonable correlation with clinic audiometry, supporting family engagement and between-visit monitoring rather than diagnosis [45].

#### Safety, Stewardship, and Workflow

Across randomized and comparative studies, smartphone otoscopy did not increase AOM diagnosis or antibiotic prescription in emergency settings compared to conventional otoscopy, and in some trainee contexts, it was associated with fewer prescriptions, likely due to better shared visualization and supervision [41,53]. Programs that integrate remote reviews into perioperative pathways have demonstrated high postoperative success rates and measurable cost savings for families in remote regions [32,54]. Collectively, these findings support protocolized tele-otoscopy within antibiotic stewardship approaches and access pathways that do not rely exclusively on family-owned smartphones, such as facilitated capture in schools, community programs, and clinics [28,29,34,61,64]. Additionally, remote hearing screening initiatives, when paired with follow-up during rehabilitation, may integrate synergistically with OM care, ultimately contributing to comprehensive pediatric ear health services [64,77-79]. In this review, reporting on governance and medicolegal arrangements was uncommon, representing a gap in implementation evidence. Implementation should include explicit consent, minimum image quality thresholds with default to in-person examination when unmet, and red-flag escalation [52,58,72]. Future studies should report on adverse events and medicolegal frameworks.

#### Equity and Access

Tele-otoscopy is particularly valuable in indigenous and rural settings with few specialists, where school-based or outreach models led by local health workers can achieve over 80% screening coverage and facilitate timely surgical intervention [28]. Jacups et al [80] reported that the telehealth model was the most cost-effective method for providing ENT surgery to children living in remote regions, primarily by reducing patient travel. However, equity implications depend strongly on implementation design, including whether capture is facilitated and whether connectivity and device requirements are minimized [28,43,50]. Not all low-burden mHealth interventions translate into short-term clinical improvements; for example, multimedia or text messaging alone did not increase clinic attendance or improve ear outcomes over 6 weeks in a high-risk remote population, despite good acceptability [70]. Digital divide factors—device availability, connectivity and bandwidth requirements, caregiver digital and health literacy, and accessibility beyond geography (including language support and cultural adaptation)—were infrequently reported or measured systematically across included studies [43,50]. Age-related constraints are also salient in pediatrics, with lower capture quality reported in children under 24 months, which can shift burden onto caregivers when programs rely primarily on home capture [43]. Care model choice has direct equity consequences. In lower-middle-income countries, implementation models pairing screening with video-otoscopy and electronic medical record capture can broaden the scope beyond hearing thresholds alone by identifying otologic pathology, even among children with normal hearing, while enabling structured follow-up workflows [69]. Hub-based or facilitated capture workflows (community-hosted capture hubs or school programs) can reduce reliance on family-owned devices and high-bandwidth connections while enabling standardized training and explicit image quality thresholds [28,29,34]. In contrast, home-based models can improve convenience but may disproportionately disadvantage families facing barriers in time, literacy, language, connectivity, or device access unless hardware and support are provisioned [43,68].

Therefore, equity-focused deployment should prioritize community-hosted capture hubs, offline-capable apps, and hardware provision [28,43,50]. In pediatrics, careful consideration of caregivers’ role is essential, as their involvement can influence capture quality and follow-through [12,65]. Importantly, few included studies conducted formal health equity analyses or reported outcomes stratified by socioeconomic status, geography, or ethnicity, limiting conclusions on differential benefits across populations and underscoring the need for equity endpoints and subgroup reporting in future evaluations [43,50].

### Limitations

This scoping review emphasized breadth rather than quantitative synthesis and therefore did not include a meta-analysis; however, we conducted a targeted QUADAS-2 appraisal for diagnostic accuracy studies. Limitations included the enrollment of mixed-age cohorts, lack of blinded reference standards, and the rapid evolution of device ecosystems, which may reduce the generalizability of device-specific findings. Additionally, language and publication restrictions may have led to the exclusion of relevant gray literature. Although we searched multiple bibliographic databases and used both controlled vocabulary and free-text terms, relevant studies may have been missed due to terminology variation across disciplines and incomplete indexing of emerging digital health tools. Future studies should prioritize large, multicenter evaluations that incorporate standardized blinded reference standards to strengthen diagnostic accuracy across diverse pediatric populations and care settings. External validation efforts must address varied device ecosystems and real-world image quality to ensure generalizability. Comparative research is warranted to establish the relative performance and cost-effectiveness of emerging digital otoscopy, acoustic, and decision support tools. This scoping review protocol was not prospectively registered. Core methodological decisions were defined a priori; minor refinements to screening guidance were made during pilot screening to improve consistency. Although scoping reviews do not always require registration, the absence of prospective registration may reduce transparency and should be considered when interpreting the findings. In addition, interpretation of diagnostic accuracy is constrained by substantial clinical and methodological heterogeneity across studies, which limits cross-setting comparability of κ, sensitivity, and specificity. Equity-relevant reporting was uncommon, restricting conclusions on differential benefits across populations. Finally, while implementation outcomes were frequently described, most studies did not evaluate sustainability or scale-up using an explicit implementation framework, and evidence for long-term maintenance remains limited.

### Future Research Agenda

Based on mapped gaps across technologies, care models, and operators, priority research directions include (1) multisite pragmatic evaluations with standardized reporting of pediatric age strata, OM phenotype, reference standards, operator training content and competency, and explicit image quality thresholds; (2) comparative evaluation of hub-based facilitated capture versus home-based capture models, including equity-relevant endpoints and subgroup analyses; (3) workflow-focused outcomes beyond accuracy; (4) sustainability and scale-up studies using an implementation framework, including maintenance, governance, and medicolegal arrangements; and (5) for artificial intelligence–enabled tools, external validation across devices or sites with real-world image quality, transparent dataset description, and prospective evaluation of clinical impact within the care pathway rather than standalone discrimination metrics.

### Conclusions

Telehealth for pediatric OM now spans mature technologies and care models. Store-and-forward tele-otoscopy is feasible and, with standardized image capture and brief structured training, can approach in-person diagnostic performance. Implementation quality requires predefined image quality thresholds and a default to in-person care when those thresholds are not met. It also requires integration into hybrid workflows and basic governance covering consent, documentation, and data protection. Programs should ensure equitable access, especially in resource-limited settings. Video-only encounters without otoscopic imaging are inadequate when decisions require objective middle ear findings. Key evidence gaps include multicenter child-level outcomes, economic evaluations—particularly in low- and middle-income countries—and external validation of artificial intelligence–based diagnostic tools across devices and sites. These priorities should guide the next phase of research and scale-up.

## Supplementary material

10.2196/85416 Multimedia Appendix 1 Full search strategies for this scoping review.

10.2196/85416 Multimedia Appendix 2 Study-level characteristics and data charting for included studies (N=52). This appendix presents the data extraction (data charting) table for all included studies in this scoping review. Variables include study design and setting, country, participant characteristics (pediatric population), otitis media phenotype, telehealth care model (asynchronous/synchronous/hybrid), device or technology category, operator or capture and interpretation workflow, training and image quality considerations, outcome domains reported, and funding or conflict of interest statements where available.

10.2196/85416 Multimedia Appendix 3 RE-AIM mapping of implementation-related outcomes and reporting gaps. This appendix summarizes implementation-relevant constructs using the Reach, Effectiveness, Adoption, Implementation, and Maintenance (RE-AIM) framework across included studies. The table highlights commonly reported elements and underreported domains to inform future research and reporting in telehealth-supported pediatric otitis media care.

10.2196/85416 Multimedia Appendix 4 Revised Quality Assessment of Diagnostic Accuracy Studies (QUADAS-2). This appendix reports the QUADAS-2 risk of bias and applicability assessments for the subset of included studies that evaluated diagnostic accuracy. Judgments are provided across QUADAS-2 domains (patient selection, index test, reference standard, flow and timing) and applicability domains (patient selection, index test, reference standard), with signaling questions and domain-level ratings.

10.2196/85416 Checklist 1 PRISMA-ScR checklist.

## Abbreviations

AOM: acute otitis media
AUC: area under the receiver operating characteristic curve
CSOM: chronic suppurative otitis media
DenseNet: Dense Convolutional Network
ENT: ear, nose, and throat
JBI: Joanna Briggs Institute
MEE: middle ear effusion
mHealth: mobile health
OM: otitis media
OME: otitis media with effusion
PCC: population, concept, and context
PRISMA-S: an extension to the PRISMA Statement for Reporting Literature Searches in Systematic Reviews
PRISMA-ScR: Preferred Reporting Items for Systematic Reviews and Meta-Analyses extension for Scoping Reviews
QUADAS-2: revised Quality Assessment of Diagnostic Accuracy Studies tool
RAOM: recurrent acute otitis media
RE-AIM: reach, effectiveness, adoption, implementation, and maintenance
ROC: receiver operating characteristic
