# Supplementary material for: Telehealth Approaches for Pediatric Otitis Media and Clinical Outcomes: Scoping Review
Source: J Med Internet Res. 2026 Mar 18;28:e85416. doi: 10.2196/85416 (PMC12999227; doi:10.2196/85416)
Supplement: Multimedia Appendix 1 [file jmir-v28-e85416-s001.docx]

**Multimedia Appendix 1. Full Search Strategies**

**PubMed（MEDLINE via PubMed）**

(

(

"Otitis Media"[Mesh] OR "Otitis Media with Effusion"[Mesh] OR "Otitis Media, Suppurative"[Mesh]

OR "otitis media"[tiab] OR otitis[tiab]

OR "acute otitis media"[tiab] OR AOM[tiab]

OR "otitis media with effusion"[tiab] OR OME[tiab]

OR "middle ear effusion"[tiab] OR MEE[tiab]

OR "glue ear"[tiab]

OR "recurrent acute otitis media"[tiab] OR RAOM[tiab]

OR "chronic suppurative otitis media"[tiab] OR CSOM[tiab]

OR "ear infection*"[tiab]

)

AND

(

"Child"[Mesh] OR "Infant"[Mesh] OR "Adolescent"[Mesh] OR "Pediatrics"[Mesh]

OR child*[tiab] OR infant*[tiab] OR newborn*[tiab] OR neonat*[tiab]

OR toddler*[tiab] OR preschool*[tiab] OR "school-age*"[tiab]

OR pediatric*[tiab] OR paediatric*[tiab]

OR adolescen*[tiab] OR teen*[tiab] OR youth*[tiab]

)

AND

(

"Telemedicine"[Mesh] OR "Remote Consultation"[Mesh] OR "Videoconferencing"[Mesh]

OR "Mobile Applications"[Mesh] OR "Cell Phone"[Mesh]

OR telemedicine[tiab] OR telehealth[tiab] OR telecare[tiab] OR "tele-care"[tiab]

OR teleconsult*[tiab] OR "remote consult*"[tiab] OR "remote visit*"[tiab]

OR "virtual visit*"[tiab] OR "video visit*"[tiab] OR televisit*[tiab]

OR "video consultation"[tiab] OR videoconferenc*[tiab]

OR "store and forward"[tiab] OR "store-and-forward"[tiab]

OR asynchronous[tiab] OR synchronous[tiab]

OR eHealth[tiab] OR mhealth[tiab] OR "mobile health"[tiab] OR "digital health"[tiab]

OR smartphone*[tiab] OR "mobile app*"[tiab]

OR teleotoscop*[tiab] OR "tele-otoscop*"[tiab] OR teleotolog*[tiab] OR "tele-otolog*"[tiab]

OR "remote otoscop*"[tiab]

OR otoscop*[tiab] OR otoscope*[tiab]

OR "video otoscop*"[tiab] OR "digital otoscop*"[tiab]

OR "smartphone otoscop*"[tiab] OR "smartphone otoscope"[tiab]

OR otoendoscop*[tiab]

OR tympanometr*[tiab] OR "wideband tympanometr*"[tiab]

OR reflectometr*[tiab] OR "acoustic reflectometr*"[tiab]

OR "Artificial Intelligence"[Mesh] OR "Machine Learning"[Mesh]

OR "artificial intelligence"[tiab] OR "machine learning"[tiab] OR "deep learning"[tiab]

OR "neural network*"[tiab] OR algorithm*[tiab] OR classifier*[tiab]

OR "computer-aided"[tiab] OR "computer assisted"[tiab]

)

)

AND english[la]

NOT (animals[mh] NOT humans[mh])

**Web of science** **Core Collection（Clarivate）**

TS=(

("otitis media" OR otitis OR "acute otitis media" OR AOM OR "otitis media with effusion" OR OME

OR "middle ear effusion" OR MEE OR "glue ear" OR "ear infection*" OR RAOM OR CSOM)

AND

(child* OR pediatric* OR paediatric* OR infant* OR newborn* OR neonat* OR toddler* OR adolescen* OR teen* OR youth* OR "school-age*" OR preschool*)

AND

(telemedicine OR telehealth OR telecare OR teleconsult* OR "remote consult*" OR "remote visit*" OR "virtual visit*" OR "video visit*"

OR videoconferenc* OR "store and forward" OR "store-and-forward" OR asynchronous OR synchronous

OR eHealth OR mHealth OR "mobile health" OR "digital health"

OR teleotoscop* OR "tele-otoscop*" OR teleotolog* OR "tele-otolog*"

OR "remote otoscop*" OR otoscop* OR otoscope*

OR "video otoscop*" OR "digital otoscop*" OR otoendoscop*

OR "smartphone otoscop*" OR "smartphone otoscope"

OR tympanometr* OR "wideband tympanometr*" OR reflectometr* OR "acoustic reflectometr*"

OR "artificial intelligence" OR "machine learning" OR "deep learning" OR "neural network*" OR algorithm* OR classifier* OR "computer-aided" OR "computer assisted")

)

Refined by: LANGUAGES=(ENGLISH))

**Scopus（Elsevier）**

TITLE-ABS-KEY(

(

"otitis media" OR otitis OR "acute otitis media" OR AOM

OR "otitis media with effusion" OR OME

OR "middle ear effusion" OR MEE

OR "glue ear"

OR "recurrent acute otitis media" OR RAOM

OR "chronic suppurative otitis media" OR CSOM

OR (ear W/1 infection*)

)

AND

(

child* OR pediatric* OR paediatric* OR infant* OR newborn* OR neonat*

OR toddler* OR preschool* OR adolescen* OR teen* OR youth*

OR ("school age" OR schoolage*)

)

AND

(

telemedicine OR telehealth OR telecare OR teleconsult*

OR (remote W/1 consult*) OR (remote W/1 visit*)

OR ("virtual visit" OR "virtual visits") OR ("video visit" OR "video visits")

OR televisit* OR (video W/1 consultation*) OR videoconferenc*

OR ("store and forward" OR "store-and-forward")

OR asynchronous OR synchronous

OR ehealth OR mhealth OR "mobile health" OR "digital health"

OR teleotoscop* OR (tele W/1 otoscop*) OR teleotolog* OR (tele W/1 otolog*)

OR (remote W/1 otoscop*) OR otoscop* OR otoscope* OR otoendoscop*

OR (video W/1 otoscop*) OR (digital W/1 otoscop*)

OR (smartphone W/1 otoscop*) OR (smartphone W/1 otoscope*)

OR tympanometr* OR (wideband W/1 tympanometr*) OR reflectometr* OR (acoustic W/1 reflectometr*)

OR "artificial intelligence" OR "machine learning" OR "deep learning"

OR "neural network*" OR algorithm* OR classifier* OR "computer-aided" OR "computer assisted"

)

)

**Cochrane** **CENTRAL**

#1 MeSH descriptor: [Otitis Media] explode all trees

#2 MeSH descriptor: [Otitis Media with Effusion] explode all trees

#3 MeSH descriptor: [Otitis Media, Suppurative] explode all trees

#4 (otitis media OR otitis OR "acute otitis media" OR AOM OR "otitis media with effusion" OR OME

OR "middle ear effusion" OR MEE OR "glue ear" OR RAOM OR CSOM OR "ear infection*"):ti,ab,kw

#5 MeSH descriptor: [Child] explode all trees

#6 MeSH descriptor: [Infant] explode all trees

#7 MeSH descriptor: [Adolescent] explode all trees

#8 (child* OR pediatric* OR paediatric* OR infant* OR newborn* OR neonat* OR toddler* OR adolescen* OR teen* OR youth* OR "school-age*" OR preschool*):ti,ab,kw

#9 MeSH descriptor: [Telemedicine] explode all trees

#10 MeSH descriptor: [Remote Consultation] explode all trees

#11 MeSH descriptor: [Videoconferencing] explode all trees

#12 MeSH descriptor: [Mobile Applications] explode all trees

#13 MeSH descriptor: [Cell Phones] explode all trees

#14 MeSH descriptor: [Artificial Intelligence] explode all trees

#15 MeSH descriptor: [Machine Learning] explode all trees

#16 (telemedicine OR telehealth OR telecare OR teleconsult* OR "remote consult*" OR "remote visit*" OR "virtual visit*" OR "video visit*"

OR videoconferenc* OR "store and forward" OR "store-and-forward" OR asynchronous OR synchronous

OR eHealth OR mHealth OR "mobile health" OR "digital health"

OR teleotoscop* OR "tele-otoscop*" OR teleotolog* OR "tele-otolog*"

OR "remote otoscop*" OR otoscop* OR otoscope*

OR "video otoscop*" OR "digital otoscop*" OR otoendoscop*

OR "smartphone otoscop*" OR "smartphone otoscope"

OR tympanometr* OR "wideband tympanometr*" OR reflectometr* OR "acoustic reflectometr*"

OR "artificial intelligence" OR "machine learning" OR "deep learning" OR "neural network*" OR algorithm* OR classifier* OR "computer-aided" OR "computer assisted"):ti,ab,kw

#17 (#1 OR #2 OR #3 OR #4) AND (#5 OR #6 OR #7 OR #8) AND (#9 OR #10 OR #11 OR #12 OR #13 OR #14 OR #15 OR #16)
